# A Semi-Supervised Adaptive Matrix Machine Approach for Fault Diagnosis in Railway Switch Machine

**DOI:** 10.3390/s24134402

**Published:** 2024-07-07

**Authors:** Wenqing Li, Zhongwei Xu, Meng Mei, Meng Lan, Chuanzhen Liu, Xiao Gao

**Affiliations:** School of Electronic and Information Engineering, Tongji University, Shanghai 201804, China; 2310877@tongji.edu.cn (W.L.); mei_meng@163.com (M.M.); 2310912@tongji.edu.cn (M.L.); liuchuanzhen88@163.com (C.L.); xiao_gao1@163.com (X.G.)

**Keywords:** fault diagnosis, semi-supervised learning, adaptive matrix machine, pseudo-labeling imbalance, switch machine

## Abstract

The switch machine, an essential element of railway infrastructure, is crucial in maintaining the safety of railway operations. Traditional methods for fault diagnosis are constrained by their dependence on extensive labeled datasets. Semi-supervised learning (SSL), although a promising solution to the scarcity of samples, faces challenges such as the imbalance of pseudo-labels and inadequate data representation. In response, this paper presents the Semi-Supervised Adaptive Matrix Machine (SAMM) model, designed for the fault diagnosis of switch machine. SAMM amalgamates semi-supervised learning with adaptive technologies, leveraging adaptive low-rank regularizer to discern the fundamental links between the rows and columns of matrix data and applying adaptive penalty items to correct imbalances across sample categories. This model methodically enlarges its labeled dataset using probabilistic outputs and semi-supervised, automatically adjusting parameters to accommodate diverse data distributions and structural nuances. The SAMM model’s optimization process employs the alternating direction method of multipliers (ADMM) to identify solutions efficiently. Experimental evidence from a dataset containing current signals from switch machines indicates that SAMM outperforms existing baseline models, demonstrating its exceptional status diagnostic capabilities in situations where labeled samples are scarce. Consequently, SAMM offers an innovative and effective approach to semi-supervised classification tasks involving matrix data.

## 1. Introduction

**Figure 1:**
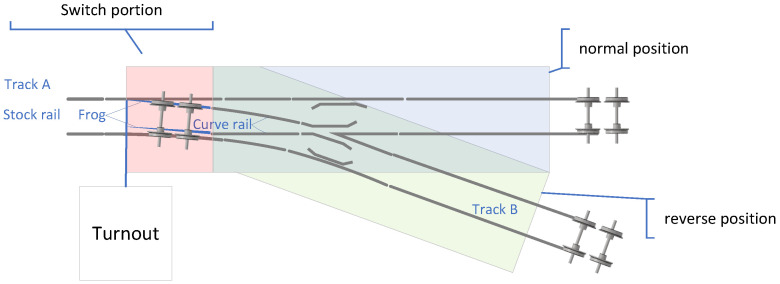
Turnout structural schematic.

The turnout represents one of the three primary railroad outdoor components, with its condition having a direct impact on the safety of shunting and train station traffic [1]. The switch machine locks the track in either the directional or the reverse direction, serving as the motion execution unit of the turnout, as shown in Figure 1. Currently, the primary maintenance approach for turnout equipment is a combination of “cycle repair” and “fault repair” [2]. Maintenance personnel obtain the action current and power data of the switch machine through the centralized signaling monitoring (CSM) system and analyze the working status of the turnouts based on their professional knowledge and experience, thereby aiding in turnout maintenance. However, the conversion signals of switch machine equipment exhibit non-stationary and non-linear characteristics. The numerous types of turnout faults with complex characteristics make fault detection and classification very challenging. This maintenance approach has several drawbacks, including long fault delays, low fault diagnosis accuracy, and high labor intensity. Additionally, it often leads to “under-maintenance” and “over-maintenance,” highlighting the limitations of the current maintenance model. Fault diagnosis of the switch machine is crucial in providing a reliability guarantee for the entire life cycle of the interlocking, attracting extensive attention from experts and scholars in the field of condition repair [3].

With the advancement of intelligent operation and maintenance of railroad electric services, an increasing number of data-driven fault diagnosis methods have emerged for switch machines and other electromechanical equipment [4,5]. These methods are categorized into three types according to their feature classification strategy: (1) distance-based, determining the abnormality by setting standard curves [6,7]; (2) classifier-based, including support vector machine (SVM) [8], k-nearest neighbor(KNN), [9] and random forest (RF) [10]; (3) deep-learning-based methods, including convolutional auto-encoder (CAE) [11], convolutional neural networks (CNN) [12], and long short-term memory networks (LSTM) [13]. However, these methods demand substantial professional and field-specific expertise, and obtaining labeled data is both laborious and expensive, hindering intelligent fault diagnosis [14,15].

Since semi-supervised learning (SSL) offers a solution to requiring only a few labeled data, numerous studies have begun incorporating SSL to enhance fault diagnosis performance [16,17]. Lao et al. [18] introduced a semi-supervised weighted prototype network (SSWPN) targeting the issue of switch machine fault diagnosis with limited labeling. Shi et al. [19] extracted dynamic current profile features, integrating SVM with semi-supervised strategies to ascertain the turnout state. In semi-supervised learning, pseudo-labeling serves as an essential strategy. Initially, the model is trained using a limited dataset of labeled examples. Subsequently, it assigns labels to a substantial volume of unlabeled data based on predictions. Predictions made with high confidence are considered accurate labels and are then integrated into the further training of the model [20]. Although some studies have introduced semi-supervision into the field of fault diagnosis, semi-supervised fault diagnosis for switch machine troubleshooting remains nascent, facing several challenges. (1) Despite training with balanced data and evaluating balanced target data, an inherent imbalance in pseudo-labels emerges due to data similarity [21]. In classification models, the loss penalty plays a crucial role in defining the boundary between two hyperplanes. Regrettably, applying a single penalty parameter across all samples results in hyperplane shifts in unbalanced class distributions, with the model penalizing minority classes and favoring the majority class for hyperplane delineation. (2) Acquired signals are inherently represented as fault images, showcasing varied correlations among the matrix’s rows and columns. However, training with solely vector features, reliant on expert knowledge, inevitably compromises the spatial feature integrity when vectorizing matrix samples.

Several scholars have embarked on studies to directly utilize matrix samples for modeling. Capturing the rank of a matrix, a measure of the correlation between its rows and columns, is crucial for constructing a matrix classifier. Various researchers have introduced diverse approaches to address the matrix rank issue, including decomposing the matrix into rank-k matrices [22,23] or constraining the matrix rank to 1 [24]. However, these techniques often necessitate predetermining the matrix rank, thus limiting their practicality. Conversely, support matrix machines (SMM) [25] suggest employing the kernel norm to approximate matrix ranks, enabling direct classification of 2D matrix features without presetting the ranks, thereby preserving the data’s structural integrity. In recent years, efforts have been made to enhance SMM’s performance through innovations like the multi-class support matrix machine (MSMM) [26], multi-class support matrix machine based on evolutionary optimization (MSMM-CE) [27], security transfer support matrix machine (STSMM) [28], among others. Recent studies have demonstrated that approximating rankings with kernel norms is suboptimal [29,30]. The total of the singular values, each of which has a size indicative of its significance, forms the nuclear norm. Using the kernel norm to approximate matrix rank treats all singular values equally and lacks adaptiveness, significantly reducing its flexibility [31].

This study introduces a semi-supervised adaptive matrix machine (SAMM) method for diagnosing faults in switch machines, specifically applying it to analyze current signals from the ZDJ9 switch machine dataset. The diagnostic capabilities of this method have been validated through experimental comparisons, which demonstrate its superiority over other contemporary diagnostic approaches. The primary contributions of this research are threefold:

(1) The incorporation of an adaptive low-rank regularizer selectively retains larger singular values, improving the approximation of the matrix rank and enhancing the extraction of fundamental connections between the rows and columns of matrix data.
(2) The development of a probabilistic output strategy for SAMM, coupled with a semi-supervised learning (SSL) framework that utilizes these outputs to assign high-confidence pseudo-labels to unlabeled samples, effectively mitigating the challenges associated with a lack of labeled data.
(3) The introduction of an adaptive penalty term to address the imbalance in pseudo-label distribution, which adjusts the hinge loss penalty coefficient based on sample quantity to counteract learning biases.

The remainder of this paper is organized as follows: Section 2 offers a concise overview of the original SMM model. Section 3 describes the proposed SAMM model and its diagnostic framework in detail. Section 4 discusses the experimental validation of the method. Section 5 concludes the paper with a summary of the findings.

## 2. Support Matrix Machine

**Figure 2:**
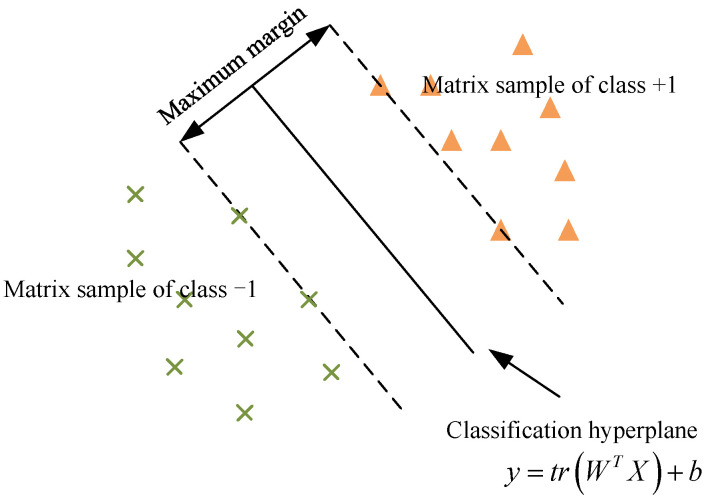
Classification principle of SMM.

The support matrix machine (SMM) is a classification methodology specifically designed to handle input data in matrix forms, shown in Figure 2. Unlike conventional classification methods, which convert matrices into vectors and potentially compromise the structural integrity of the data, SMM preserves the matrix format. This retention enables SMM to fully utilize the structural information within matrices. It introduces a novel penalty term, the spectral elastic net, to leverage this advantage. By maintaining the matrix structure, SMM effectively captures the inherent structure and correlations of the data, thereby enhancing classification performance.

The objective function of SMM is formulated as follows:

(1) $$\min_{W,b}\;\frac{1}{2}tr\left(W^{T}W\right)+\beta {\left\|W\right\|}_{*}+\rho \underset{i=1}{\sum^{N}}\;{\left\{1-t_{i}\left[tr\left(W^{T}A_{i}\right)+b_{i}\right]\right\}}_{+}$$

where $A_{i}\in R^{p\times q},i=1,2,\ldots ,N$ represents the training matrix data. $y_{i}\in \left\{-1,1\right\}$ denotes the corresponding labels. The regression matrices $W\in R^{p\times q}$ are inversely proportional to the distance of the hyperplane margin. *b* signifies the bias. β and ρ are two hyperparameters.

The objective function of the support matrix machine (SMM) comprises two principal components: the matrix-form hinge loss and the spectral elastic net penalty. Hinge loss, a common feature in classification models, promotes sparsity and robustness. In SMM, this loss function measures the classification error by calculating the discrepancy between the model’s predictions and the actual labels for each training sample. The spectral elastic net penalty, integral to SMM, exploits the structural information of the feature matrix, capturing correlations within its columns and rows. In particular, $tr\left(W^{T}W\right)$ is employed to keep the model’s complexity under control and avoid overfitting, ensuring that the model adheres to the rule of minimizing structural risk. The kernel norm ${\left\|W\right\|}_{*}$, defined as the total of the singular value decompositions of the matrix, serves as a metric for assessing the matrix’s low rank, which facilitates the extraction of structural information from matrix data [32]. However, minimizing the kernel norm by substituting the rank function with an approximation may compromise accuracy, especially in matrices with complex structures. The kernel norm represents only a relaxed approximation of the rank function, and it does not fully capture the matrix’s true rank. Significant deviations from the true rank occur within the kernel norm when the singular values differ from 1 [33]. This relaxation causes the kernel norm to over-penalize the matrix, potentially yielding a suboptimal low-rank approximation.

## 3. Semi-Supervised Adaptive Matrix Machine

In this section, the proposed semi-supervised adaptive matrix machine (SAMM) method is presented, which aims to address the challenges of insufficient labeled samples and pseudo-label imbalance, as well as to more accurately capture the correlations between matrix rows and columns. To resolve the non-smooth optimization issue within the SAMM model, an alternating update strategy is employed [34], facilitating the efficient solution of the model and the attainment of the optimal solution.

### 3.1. SAMM Model

**Figure 3:**
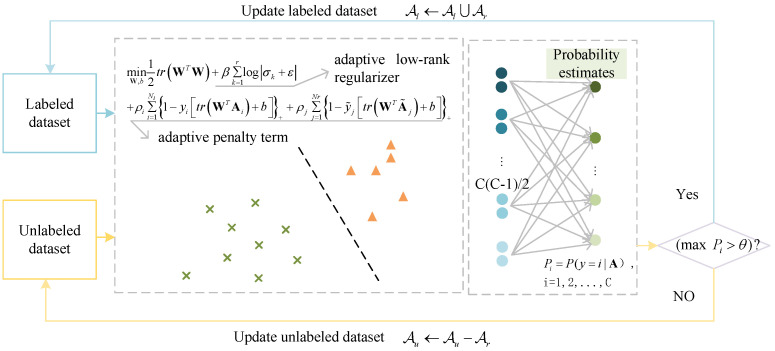
SAMM model.

The semi-supervised adaptive matrix machine (SAMM) combines semi-supervised learning with adaptive techniques, utilizing an adaptive low-rank regularizer to identify correlation within matrix data and an adaptive penalty term to mitigate the impact of inter-class samples on hyperplane margins, as shown in Figure 3. By integrating probabilistic output, which incrementally expands the labeled sample set, SAMM not only gradually improves classification performance but also autonomously adjusts model parameters during the learning phase to suit various data distributions and feature architectures. The objective function for SAMM is delineated as follows:

(2) $$\begin{array}{c} \min_{W,b}\;\frac{1}{2}tr\left(W^{T}W\right)+\beta \underset{k=1}{\sum^{r}}\;\log\left|\sigma_{k}+\varepsilon \right|\\ +\rho_{i}\underset{i=1}{\sum^{N_{l}}}\;{\left\{1-y_{i}\left[tr\left(W^{T}A_{i}\right)+b\right]\right\}}_{+}+\rho_{j}\underset{j=1}{\sum^{Nr}}\;{\left\{1-{\tilde{y}}_{j}\left[tr\left(W^{T}{\tilde{A}}_{j}\right)+b\right]\right\}}_{+} \end{array}$$

The first term influences the model in the same way as observed in the support matrix machine (SMM). The second term $\beta \sum_{k=1}^{r}\;\log\left|\sigma_{k}+\varepsilon \right|$ introduces an adaptive low-rank regularizer, where ε is a sufficiently small positive number, ensuring $\sigma_{k}+\varepsilon$ is not zero. Larger singular values correspond to row and column information within the matrix and should be preserved, whereas smaller singular values, which are often linked to irrelevant or redundant data, should be discarded. Adaptive low-rank regularizers maintain these larger singular values and reduce the smaller ones to zero or near-zero values. By minimizing this regularizer, SAMM effectively extracts low-rank matrix information and adaptively selects and preserves singular values associated with highly correlated data. Utilizing the adaptive low-rank regularizer allows SAMM to more accurately estimate the matrix’s rank and extract strong correlations between rows and columns from the matrix data. This adaptivity enables SAMM to handle matrix-form data more effectively and improve its classification performance.

The adaptive penalty term computes category weights according to the ratio of pseudo-labeled samples per category, adjusting the hinge loss’s penalty parameter $\rho_{i}$ based on these weights to optimize the handling of unbalanced samples. If samples are balanced, the penalty remains constant $\rho_{i}=\rho$; for unbalanced samples, $\rho_{i}$ can be determined by

(3) $$\rho_{i}=\left\{\begin{array}{c} \rho \frac{N_{2}}{N},i\in N_{1}\\ \rho \left(1+\frac{N_{1}}{N}\right),i\in N_{2} \end{array}\right.$$

N1 and N2 represent the number of samples in the majority and minority categories, respectively, while *N* denotes the combined total of samples from both categories. With the introduction of an adaptive penalty term, samples from the majority categories incur a lower penalty than those from the minority categories. Consequently, the SAMM model effectively considers the features of all categories within unbalanced datasets, thereby avoiding the problem of overemphasizing the majority categories while neglecting the minority ones.

To address the challenge of recognizing switch machine fault status with a limited number of labeled samples, we leverage both a small set of labeled and a substantial pool of unlabeled samples. We have developed a semi-supervised model that integrates probabilistic outputs, utilizing the SAMM model. Initially, the model undergoes training with the labeled dataset $A_{l}={\left\{A_{i},y_{i}\right\}}_{i=1}^{N_{l}}$. The Platt Scaling [35] method is utilized to map the output of the SAMM model for each sample into the [0, 1] interval, serving as a probability estimate of the sample’s category membership. Utilizing the Wu method [36], we couple C(C−1)/2 SAMM probability estimates pairwise into a single value, wherein the maximum probability output indicates the unlabeled sample’s confidence level for true category membership. Confidence thresholding is a widely used technique to enhance pseudo-labeling. By setting a higher threshold, the reliability of pseudo-labels is improved [37]. A large unlabeled dataset $A_{u}={\left\{A_{j}\right\}}_{j=1}^{N_{u}}$ is provided, from which samples that exceed a specified confidence threshold are incorporated into the labeled dataset as reliable samples $A_{r}={\left\{{\tilde{A}}_{j},{\tilde{y}}_{j}\right\}}_{j=1}^{N_{r}}$. Repeat this process until no samples exceed the confidence threshold or the maximum iteration count is achieved. This semi-supervised learning approach effectively addresses the switch machine fault diagnosis challenge with few labeled samples, diminishing the time and economic expenditures associated with sample labeling.

### 3.2. SAMM Learning Algorithm

Solving the SAMM model presents a non-smooth optimization challenge, complicating the search for a globally optimal solution. To address this issue, the alternating direction multiplier method (ADMM) is introduced as an effective algorithm for the SAMM model’s resolution. The ADMM algorithm tackles the original challenge by breaking it down into two subproblems and applying iterative alternating updates. During each iteration, the ADMM algorithm incrementally approaches the optimal solution by updating primal and dual variables. This iterative procedure efficiently resolves the SAMM model, achieving an optimal solution characterized by low rank and sparsity. In the ADMM framework, Equation (2) is reformulated as Equation (4).

(4) $$\begin{array}{cc} \underset{W,b,Z}{\arg\min}\; & h(W,b)+g(Z)\\ \;\;\;\;\;\;\;s.t. & Z-W=0 \end{array}$$

here $h\left(W,b\right)=\frac{1}{2}tr\left(W^{T}W\right)+\rho_{i}\sum_{i=1}^{N_{l}}\;{\left\{1-y_{i}\left[tr\left(W^{T}A_{i}\right)+b\right]\right\}}_{+}+\rho_{j}\sum_{j=1}^{Nr}\;{\left\{1-{\tilde{y}}_{j}\left[tr\left(W^{T}{\tilde{A}}_{j}\right)+b\right]\right\}}_{+}$$g\left(Z\right)=\tau \sum_{k=1}^{r}\;\log\left|\sigma_{k}+\varepsilon \right|$$\sigma_{k}$ is the kth singular value of the matrix Z, k=1,2,...,r

The augmented Lagrangian function is subsequently defined as follows

(5) $$L\left(W,b,Z,\Lambda \right)=h\left(W,b\right)+g\left(Z\right)+\langle \Lambda ,Z-W\rangle +\frac{\delta }{2}{\left\|Z-W\right\|}_{F}^{2}$$

δ>0 represents the step size, and Λ denotes the Lagrange multiplier. Following the ADMM framework, the objective function is divided into two subproblems (concerning Z and W) and resolved through iterative computation. During each iteration, the solver sequentially minimizes Z and W, followed by an update to the Lagrange multipliers in alignment with these adjustments. W, Z, and Λ are updated as follows.

(6) $$\left\{\begin{array}{c} Z^{\left(t+1\right)}=\arg\min L\left(Z,W^{\left(t\right)},\Lambda^{\left(t\right)}\right)\\ W^{\left(t+1\right)}=\arg\min L\left(Z^{\left(t+1\right)},W,\Lambda^{\left(t\right)}\right)\\ \Lambda^{\left(t+1\right)}=\Lambda^{\left(t\right)}+\delta \left(Z^{\left(t+1\right)}-W^{\left(t+1\right)}\right) \end{array}\right.$$

Here t and t + 1 signify the tth and t + 1th iterations, respectively.

(1) To solve the subproblem of Z, assume (W,b) and Λ are held constant, reducing it to a function concerning Z expressed as:
  (7) $$\begin{array}{cc} L_{Z}= & g\left(Z\right)+\langle \Lambda ,Z-W\rangle +\frac{\delta }{2}{\left\|Z-W\right\|}_{F}^{2}\\ = & g\left(Z\right)+\frac{\delta }{2}{∥Z-W+\frac{\Lambda }{\delta }∥}_{F}^{2} \end{array}$$

Let $I=W-\frac{\Lambda }{\delta }$ undergo singular value decomposition (SVD) in the following manner: $I=U_{I}\Sigma_{I}V_{I}^{T}$. According to [31], Z can be solved as

(8) $$Z^{*}=U_{I}Prox_{\tau }\left(\Sigma_{I_{k}}\right)V_{I}^{T}$$

where the nearest neighbor operator

$$Prox_{\tau }\left(\sigma_{i}\right)=\left\{\begin{array}{c} \max\left(\sigma_{i}-\frac{2\tau }{\sigma_{i}+\sqrt{\sigma_{i}^{2}-4\tau }},0\right)\\ 0,i\in I_{2} \end{array}\right.,i\in I_{1}$$

here $I_{1}=\left\{i|\sigma_{i}^{2}-4\tau \geq 0\right\},\;I_{2}=\left\{i|\sigma_{i}^{2}-4\tau <0\right\}$

(2) To address the subproblem concerning W, we undertake minimization of the expression encapsulating all terms associated with W as outlined in Equation (5).

(9) $$L_{W}=h\left(W,b\right)+\langle \Lambda ,Z-W\rangle +\frac{\delta }{2}{\left\|Z-W\right\|}_{F}^{2}$$

assume

(10) $$\begin{array}{c} \begin{array}{c} \min_{W,b}\;\frac{1}{2}tr\left(W^{T}W\right)+\sum_{i=1}^{N}\;\rho_{i}\xi_{i}+\langle \Lambda ,Z-W\rangle +\frac{\delta }{2}{\left\|Z-W\right\|}_{F}^{2}\\ s.t.y_{i}\left[tr\left(W^{T}A_{i}\right)+b\right]\geq 1-\xi_{i} \end{array}\\ \xi_{i}\geq 0,i=1,2,\ldots ,N \end{array}$$

Constructed via the Lagrange multiplier method

(11) $$\begin{array}{cc} L\left(W,b,\xi ,\alpha ,\beta \right) & =\frac{1}{2}tr\left(W^{T}W\right)+\underset{i=1}{\sum^{N}}\;\rho_{i}\xi_{i}+\langle \Lambda ,Z-W\rangle +\frac{\delta }{2}{\left\|Z-W\right\|}_{F}^{2}\\ & -\underset{i=1}{\sum^{N}}\;\alpha_{i}\left\{y_{i}\left[tr\left(W^{T}A_{i}\right)+b\right]-1+\xi_{i}\right\}-\underset{i=1}{\sum^{N}}\;\beta_{i}\xi_{i} \end{array}$$

with partial derivatives set to zero for *b* and $\beta_{i}$.

(12) $$\left\{\begin{array}{c} \frac{\partial L}{\partial b}=\underset{i=1}{\sum^{N}}\;\alpha_{i}y_{i}=0\\ \frac{\partial L}{\partial \xi_{i}}=\underset{i=1}{\sum^{N}}\;\rho_{i}-\underset{i=1}{\sum^{N}}\;\beta_{i}=0 \end{array}\right.$$

Upon substituting Equation (12) into Equation (11), we obtain:

(13) $$\begin{array}{cc} L\left(W,b,\xi ,\alpha ,\beta \right) & =\frac{1}{2}tr\left(W^{T}W\right)+\langle \Lambda ,Z-W\rangle +\frac{\delta }{2}{\left\|Z-W\right\|}_{F}^{2}\\ & -\underset{i=1}{\sum^{N}}\;\alpha_{i}\left\{y_{i}\left[tr\left(W^{T}A_{i}\right)+b\right]-1\right\} \end{array}$$

Equation (13) results from differentiating concerning W and setting the derivative to zero, yielding:

(14) $$W^{*}=\frac{1}{\delta +1}\left(\Lambda +\delta Z+\underset{i=1}{\sum^{N}}\;\alpha_{i}y_{i}A_{i}\right)$$

By reinserting Equations (12) and  (14) into Equation (10), we derive the optimization problem for α as follows:

(15) $$\begin{array}{cc} \max_{\alpha }\; & -\frac{1}{2}\alpha^{T}R\alpha +r^{T}\alpha \\ \;\;\;\;\;\;\;s.t. & \underset{i=1}{\sum^{N}}\;\alpha_{i}y_{i}=0\\ & 0\leq \alpha_{i}\leq \rho_{i},i=1,2,\ldots ,N \end{array}$$

Here $R=\left[R_{ij}\right]\in R^{p\times q}$, $r=\left[r_{i}\right]\in R^{p}$

(16) $$\left\{\begin{array}{c} R_{ij}=\frac{y_{i}y_{j}tr\left(A_{i}^{T}A_{j}\right)}{\delta +1}\\ r_{i}=1-\frac{y_{i}tr\left[{\left(\Lambda +\delta Z\right)}^{T}A_{i}\right]}{\delta +1} \end{array}\right.$$

The optimal value for *b* is determined by defining an average solution as specified in

(17) $$b^{*}=\frac{1}{\left|Z^{*}\right|}\sum_{i\in S^{*}}\;\left\{y_{i}-tr\left[{\left(W^{*}\right)}^{T}A_{i}\right]\right\}$$

here $Z^{*}=\left\{i:0<\alpha_{i}<C\right\}$

Algorithm 1 outlines the proposed learning algorithm for SAMM.
**Algorithm 1:** The learning algorithm for SAMM**Input:** Training set ${\left\{A_{i},y_{i}\right\}}_{i=1}^{N}$, low-rank coefficient β, loss penalty coefficient ρ, step size δ.**Output** W, *b*1. Initialize. $W^{\left(0\right)}=0,Z^{\left(0\right)}=0,{\;\;\;\Lambda \;\;\;}^{\left(0\right)}=0,k=1$**While** not converging do2. Update $Z^{k}$ with Equation (8)3. Update $W^{k}$ and b with Equations (14) and  (17)4. Update $\Lambda^{\left(k\right)}$ with Equation (6)5. k=k+1**End**6. **Return** W, *b*

### 3.3. Fault Diagnosis Framework

**Figure 4:**
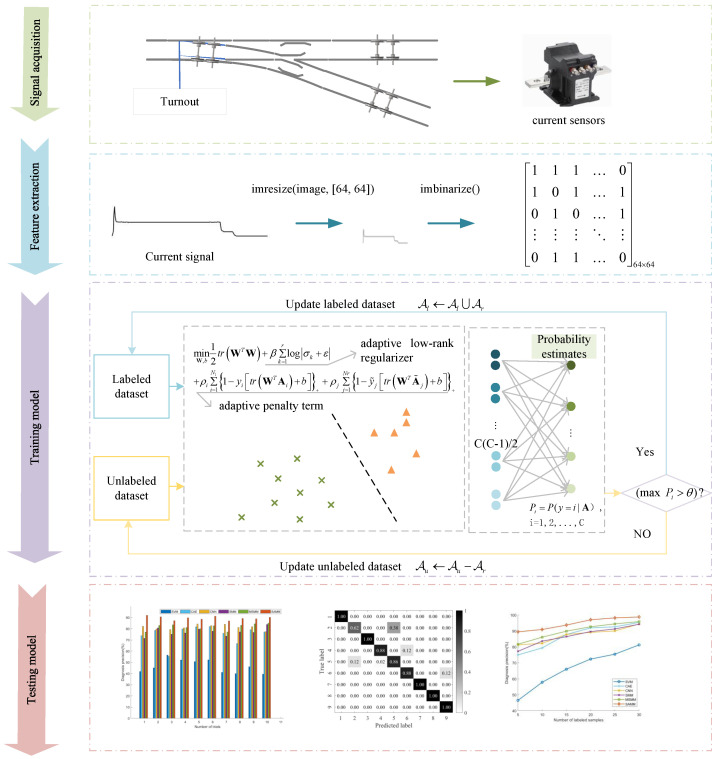
Entire framework of the proposed fault diagnosis approach.

The comprehensive framework of the model proposed herein is depicted in Figure 4, with the principal steps summarized as follows:

Step 1: Signal acquisition. Acquire current signals of the switch machine across various fault states.
Step 2: Feature extraction. Convert continuous current signals into 2D matrix samples via downsampling and binarization techniques, enabling efficient processing and model training.
Step 3: Train the SAMM Model. Labeled and unlabeled samples from the training dataset are used to build the SAMM model. The model integrates an adaptive low-rank regularizer with an adaptive penalty term, enhancing matrix structure information extraction, and addressing the pseudo-labeling imbalance challenge of semi-supervised learning.
Step 4: Test the SAMM Model. Predict the switch machine’s fault status by inputting test samples into the SAMM model.

## 4. Experimental and Discussion

### 4.1. Description of the Data Set

**Figure 5:**
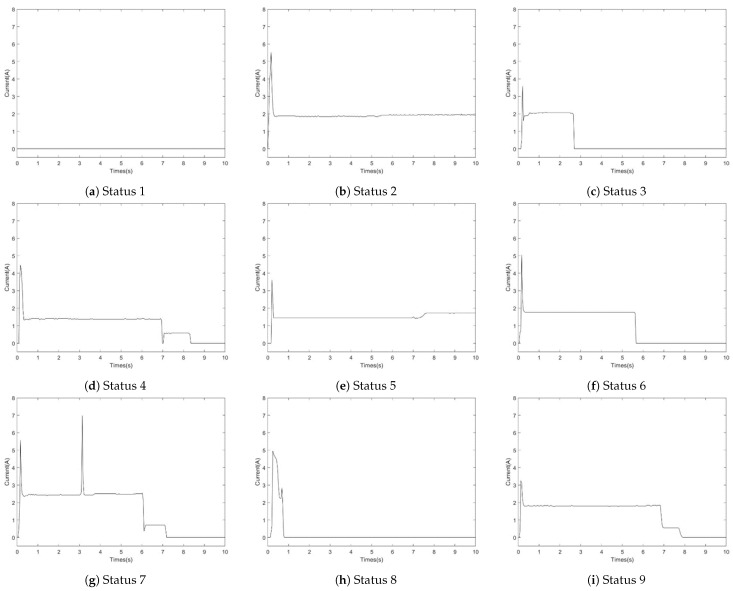
Fault status current curves of ZDJ9 switch machine.

The dataset originates from current signals generated by ZDJ9-type switch machines within the urban subway system. The SAMM model leverages current signals for fault diagnosis due to their direct correlation with the operational status of the railway switch machine. Although vibration and sound signals are also used in switch machine fault diagnosis [38,39], they present challenges in data collection and interpretation due to environmental noise and the need for precise sensor placement. Current signals, on the other hand, can be obtained through the CSM system, ensuring they are readily available and less susceptible to external noise. This method ensures minimal disruption to the switch machine’s operation. This study’s dataset was compiled by CASCO, a professional rail transit control system integrator, at specific stations along Shanghai Metro Line 13. The ZDJ9 switch machine uses a 380 V three-phase AC power supply, with phase currents A, B, and C supplying essential electrical power. This model completes a full state change in approximately 7 to 9 s, with current signals sampled at 25 Hz throughout this duration. A typical current profile encompasses four principal phases: unlocking, transition, locking, and slow release. For this study, the A-phase current curve was selected for dataset construction due to its comprehensive representation of the switch machine’s motion. As detailed in Table 1 and Figure 5, the A-phase current dataset spans nine distinct fault statuses, comprising eight fault states and one normal state. Throughout the experiment, labeled training samples per fault status varied from 5 to 30, unlabeled training samples from 45 to 25, with a constant 50 test samples. Employing down sampling and binarization techniques, each raw current signal image was transformed into a 64 × 64-dimensional feature matrix, facilitating further processing and model training.

### 4.2. Comparison Experiment

To optimize the classification performance of the SAMM model, three key parameters were precisely adjusted in the experiments: the low-rank coefficient β, the loss penalty coefficient ρ, and the step size δ. In the experiment, δ was set to 0.01. A 5-fold cross-validation method was utilized to select the structural parameters β and ρ from the set $\left\{2^{-5},2^{-4.5},\cdots ,2^{5}\right\}$, and the confidence threshold θ was set within the range of {0.5,0.55,⋯,0.95}. To guarantee fairness and comparability in our experimental outcomes, structural parameters for each model were optimized before undertaking fault diagnosis tasks, ensuring optimal operation across differing models. The identical parameter optimization process was applied to other comparative models, notably support vector machines (SVM), support matrix machines (SMM), and multi-class support matrix machines (MSMM). The models’ optimal parameters were determined using a 5-fold cross-validation technique, and the structural parameters’ value ranges were determined by consulting relevant literature. Structural parameters for deep learning models such as the convolutional auto-encoder (CAE) and convolutional neural network (CNN) were selected based on insights gleaned from relevant literature. All diagnostic models operated within a Windows 11 (64-bit) and Matlab 2023a software environment. The utilized PC’s hardware configuration primarily included an Intel(R) Core(TM) i7-13700H CPU and 32.0 GB RAM.

To thoroughly assess the classification performance across various classifiers, three evaluation metrics were employed: precision rate, recall rate, and F1 score. The precision rate quantifies the proportion of accurately identified positive class samples among those deemed to be in a positive class, reflecting the classifier’s accuracy. Conversely, recall gauges the proportion of all correctly identified samples within the actually positive class, indicating the classifier’s coverage. The F1 score, a harmonized mean of precision and recall rates, serves as a singular comprehensive metric for gauging the classifier’s overall effectiveness. Precision, recall, and F1 score, metrics suited for binary classification, were computed for each category using a macro-averaging approach and then averaged. For multicategory classification, these metrics are generalized from those utilized for k-category classification, as delineated in Table 2, and are defined as follows.

(18) $$\begin{array}{cc} \; & \;\;\;\;\;\;\;\mathrm{precision}=\frac{1}{k}\underset{c=1}{\sum^{k}}\;\frac{tp_{c}}{tp_{c}+fp_{c}},\\ \; & \;\;\;\;\;\;\;\mathrm{recall}=\frac{1}{k}\underset{c=1}{\sum^{k}}\;\frac{tp_{c}}{tp_{c}+fn_{c}},\\ \; & F_{1}=\frac{2\cdot \mathrm{precision}\cdot \mathrm{recall}}{\;\;\;\;\;\;\;\mathrm{precision}+\mathrm{recall}} \end{array}$$

where ${tp}_{c}$, ${fp}_{c}$, ${fn}_{c}$, ${tn}_{c}$ are true positives, false positives, false negatives, and true negatives within category c.

**Figure 6:**
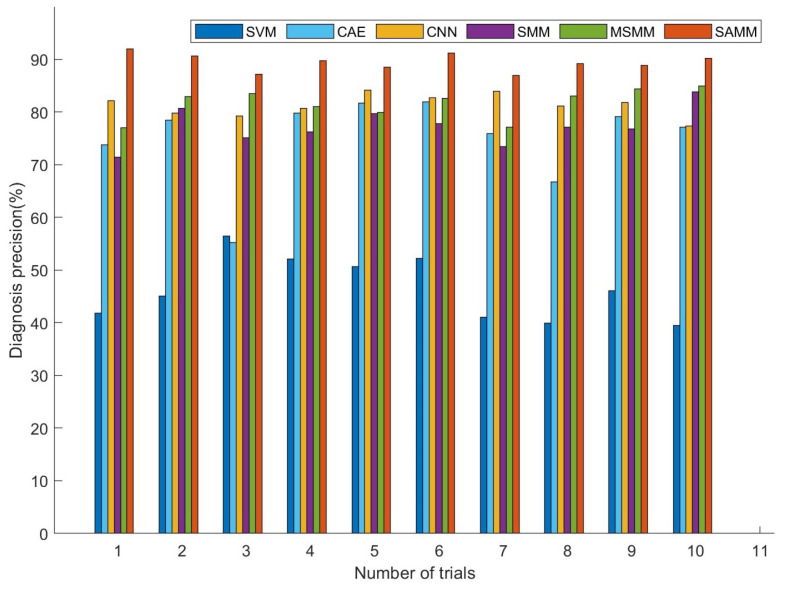
Repeat 10 times with 5 labels.

To guarantee the results’ reliability, each method was replicated 10 times for every sample case. Repeating the experiments aids in mitigating bias from random factors, thereby enhancing the robustness and credibility of the outcomes. Figure 6 displays the fault diagnosis precision for each model with merely five labeled training samples, showcasing that the SAMM model consistently outperforms others in terms of diagnostic precision across all experiments. The results demonstrate that the SAMM model sustains high diagnostic precision, even with a scarce quantity of labeled samples. Comparative results between SVM and matrix learning models (SMM, MSMM) illustrate that leveraging the structural information of images indeed enhances fault diagnosis performance. Given that SAMM adaptively leverages image structural information and mitigates the challenge of insufficient labeled samples, its overall diagnostic efficacy significantly surpasses that of the comparative models.

**Figure 7:**
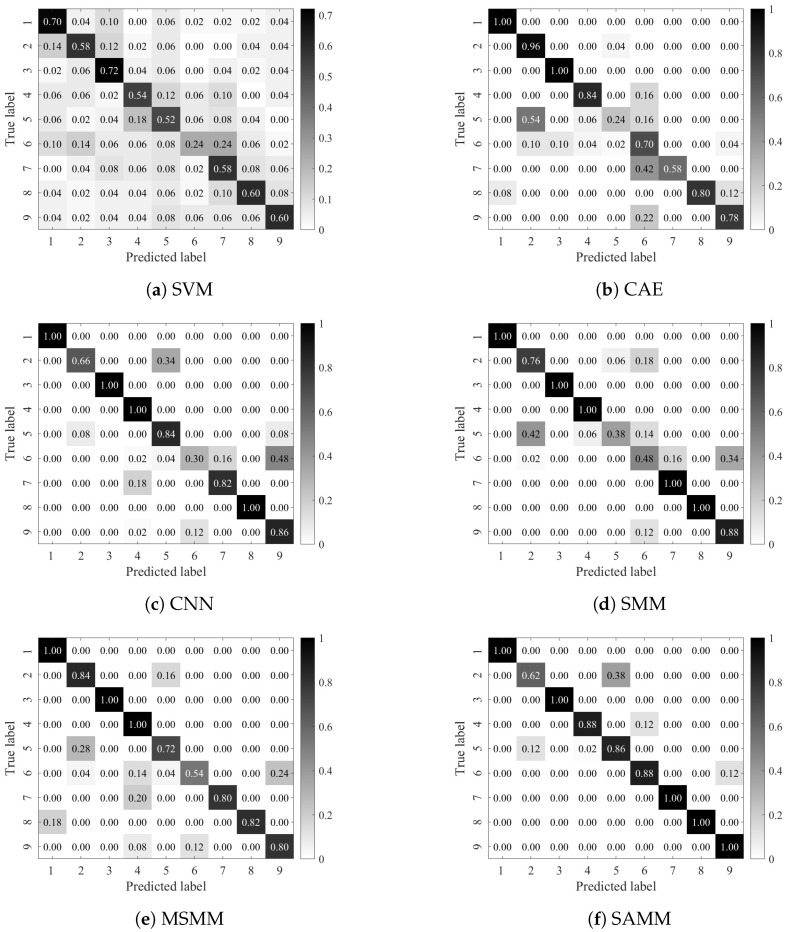
Confusion matrix of the optimal results for each model.

Figure 7 presents the confusion matrix for the optimal diagnostic outcomes across each model. The confusion matrix reveals that the SAMM model excels in identifying various fault statuses. The highest diagnostic accuracies achieved by SVM, CAE, CNN, SMM, MSMM, and SAMM are 56.45%, 82.00%, 84.15%, 83.75%, 85.00%, and 92.02%, respectively. The traditional SVM model’s diagnostic accuracy significantly trails behind other models due to its inability to fully leverage image data’s structural information. Despite the deep-learning-based CAE and CNN models’ capability to extract higher-order image features, the scarcity of labeled samples limits their accuracy from reaching the desired level. By harnessing the structural features of image data, the matrix learning models SMM and MSMM outperform CAE and CNN, albeit with certain limitations. Conversely, the SAMM model adeptly extracts low-rank structural information from matrix samples and addresses category imbalance with adaptive penalty terms, achieving a leading diagnostic accuracy of 92.02%. It showcases superior fault diagnosis performance, even with a limited number of labeled samples. This underscores the SAMM model’s advantages and efficacy in recognizing switch machine status.

**Figure 8:**
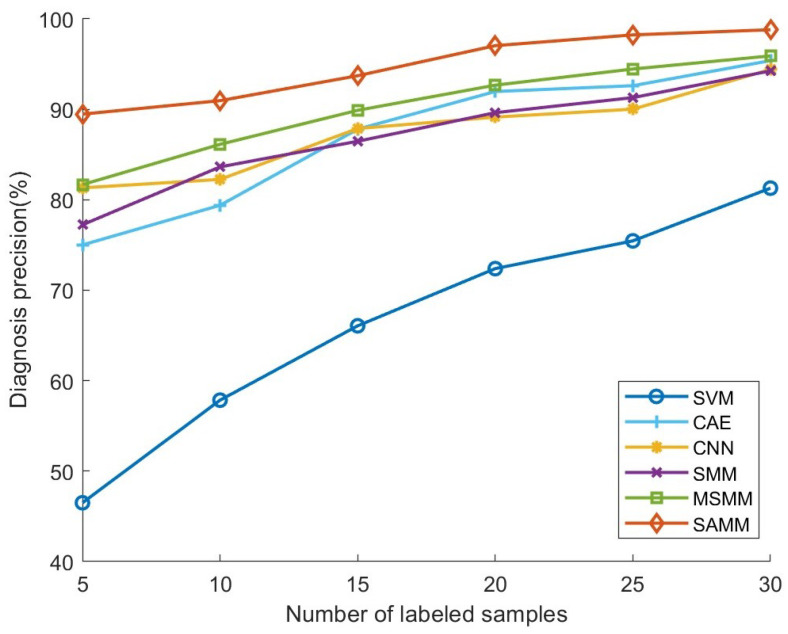
Fault diagnosis precision under different labeled samples.

Figure 8 illustrates the fault diagnosis accuracy for each model across varying counts of labeled training samples. The figure demonstrates that the diagnostic accuracy for all models improves to varying extents with an increase in labeled training samples, aligning with the inherent reliance of machine learning models on the volume of training data. Significantly, the SAMM model’s diagnostic accuracy surpasses that of the comparative models in every instance. Notably, across 5, 10, 15, 20, 25, and 30 labeled samples per fault status, the SAMM model achieved average diagnostic accuracies of 89.47%, 90.96%, 93.71%, 97.04%, 98.23%, and 98.80%, respectively, outperforming the lower accuracies recorded by the other models. The SAMM model’s exceptional diagnostic performance is credited to its utilization of an ADMM-based solver, facilitating stable convergence to the global optimum and maximizing the model’s potential. Crucially, SAMM’s integration of an adaptive low-rank regularizer with an adaptive penalty term enables precise extraction of intrinsic low-rank structural information from matrix data and effectively addresses the prevalent issue of category imbalance in semi-supervised learning. Experimental findings indicate that SAMM’s adaptive semi-supervised learning approach is particularly effective with a limited number of labeled samples. Remarkably, even with as few as five labeled samples, SAMM achieves a diagnostic accuracy of 89.47%, whereas other models exhibit a significant decline in performance. This affirms SAMM’s superiority and practical utility in addressing the challenge of scarce labeled samples.

Table 3 and Table 4 detail the recall and F1 scores, respectively, for each model across varying numbers of labeled samples. When combined with the precision outcomes previously analyzed (Figure 8), a comprehensive evaluation of the models’ overall diagnostic performance is facilitated. The recall and F1 score outcomes reveal that the SAMM model consistently outperforms all comparison models across various counts of labeled samples. With an increase in the number of labeled samples, while the recall and F1 scores for all models improve, the SAMM model’s lead persists. Integrating the experimental findings of precision, recall, and F1 score, it becomes evident that the SAMM model’s diagnostic efficacy surpasses that of other comparative models under scenarios with a limited number of labeled samples. This suggests that the strategies of employing an adaptive low-rank regularizer and adaptive penalty term enable the SAMM model to effectively tackle the challenges of scarce labeled samples and category imbalance, thereby demonstrating robust semi-supervised learning (SSL) capabilities.

The experimental outcomes comprehensively illustrate that the SAMM model optimally utilizes the intrinsic structural information of image data. Concurrently, it addresses the challenges of scarce labeled samples and category imbalance through a semi-supervised learning strategy and adaptive mechanisms, culminating in superior performance in switch machine fault diagnosis compared to other models.Despite measurement noise and interference in real-world conditions, our method has demonstrated excellent fault diagnosis capabilities in experimental validations. The results indicate that, even with some noise and interference, the SAMM method consistently achieves high accuracy in identifying and diagnosing faults in the switch machine. This underscores the practical applicability of the SAMM model in real-world scenarios.

## 5. Conclusions

This study proposes the Semi-Supervised Adaptive Matrix Machine (SAMM) model tailored to address switch machine fault diagnosis. The SAMM model features an adaptive low-rank regularizer for precise extraction of highly correlated low-rank information from matrix data and for identifying correlations between the matrix’s row and column. It employs a semi-supervised learning framework that incrementally assigns pseudo-labels to unlabeled samples based on high-confidence probabilistic outputs, thereby effectively leveraging unlabeled data. An adaptive penalty term is introduced to adjust the loss penalty in response to imbalances in category sample sizes, preventing the model from being overly biased towards the majority class. Experimental validations on the switch machine current signal dataset illustrate that SAMM surpasses other baseline models in fault diagnosis accuracy. The integration of the adaptive low-rank regularizer and adaptive penalty term effectively discerns the matrix data’s inherent structure. Concurrently, the semi-supervised framework augments training data through pseudo-labeling, yielding commendable classification outcomes, even with limited labeled samples.

In practical applications, the SAMM method significantly enhances railway switch machine fault diagnosis through the analysis of current signals recorded by the CSM system. This enables preventive maintenance, reduces dependency on extensive labeled datasets, lowers maintenance costs and time, and improves diagnostic accuracy by minimizing false alarms and missed detections. Additionally, the real-time monitoring capabilities of the CSM system, combined with the SAMM method, facilitate quick response to faults, thereby reducing fault handling time and ensuring the continuity and safety of railway operations.

In future research, we will focus on vibration and sound signals to explore new approaches for multimodal fault diagnosis, aiming to leverage the advantages of integrating multiple sensors. We will also investigate variations of the adaptive low-rank regularizer and extend SAMM’s application to fault diagnosis and anomaly detection across diverse fields.

## Figures and Tables

**Table 1:** Fault status phenomena and causes of ZDJ9 switch machine.

Label	Fault Phenomenon	Fault Cause
1	Consistently no current	Action circuit malfunction
2	The current remains constant during release	Mechanical resistance encountered
3	A sudden drop in current to zero	Insufficient contact or unlocked
4	The current release time is delayed	Abnormal motor condition
5	Increase in current during release.	Internal jamming and friction increase
6	Release without small steps	Abnormality in the indicated circuit
7	Pulse observed during switching	Poor contact of automatic switch
8	The curve only maintains 0∼1 s	Phase failure in the starting circuit
9	Normal state	Normal

**Table 2:** Binary classification confusion matrix.

Data Class	Classified as Pos.	Classified as Neg.
pos	true positive (tp)	false negative (fn)
neg	false positive (fp)	true negative (tn)

**Table 3:** Recall rate of Different Models with Varying Number of Labeled Samples.

Model	Number of Labeled Samples in Each Status					
	5	10	15	20	25	30
SVM	40.69%	54.67%	65.11%	68.22%	71.78%	80.44%
CAE	72.71%	77.33%	87.11%	91.78%	91.56%	95.33%
CNN	79.25%	80.89%	85.78%	88.00%	88.00%	94.22%
SMM	74.89%	82.22%	84.67%	89.33%	89.33%	93.56%
MSMM	79.78%	82.00%	83.78%	92.44%	94.44%	95.11%
SAMM	87.91%	90.44%	93.56%	96.89%	98.22%	98.56%

**Table 4:** F1 score of different models with varying numbers of labeled samples.

Model	Number of Labeled Samples in Each Status					
	5	10	15	20	25	30
SVM	43.25%	56.21%	65.59%	70.25%	73.57%	80.87%
CAE	73.83%	78.35%	87.46%	91.88%	92.08%	95.36%
CNN	80.24%	81.57%	86.81%	88.57%	89.00%	94.32%
SMM	76.04%	82.92%	85.56%	89.48%	90.31%	93.90%
MSMM	80.57%	84.01%	86.73%	92.56%	94.45%	95.51%
SAMM	89.47%	90.70%	93.64%	96.96%	98.23%	98.68%

## Data Availability

The data presented in this study are available on request from the corresponding author. The data are not publicly available due to privacy.

## Abbreviations

The following abbreviations are used in this manuscript:

SVM	Support vector machine
CAE	Convolutional autoencoder
CNN	Convolutional neural network
SMM	Support matrix machine
MSMM	Multiclass support matrix machine
